# Differentially Expressed Androgen-Regulated Genes in Androgen-Sensitive Tissues Reveal Potential Biomarkers of Early Prostate Cancer

**DOI:** 10.1371/journal.pone.0066278

**Published:** 2013-06-28

**Authors:** Dogus Murat Altintas, Nathalie Allioli, Myriam Decaussin, Simon de Bernard, Alain Ruffion, Jacques Samarut, Virginie Vlaeminck-Guillem

**Affiliations:** 1Institut de Génomique Fonctionnelle de Lyon (IGFL), Université de Lyon, Université Lyon, CNRS UMR5242, INRA1288, Ecole Normale Supérieure de Lyon, Lyon, France; 2 Institut des Sciences Pharmaceutiques et Biologiques (ISPB) de Lyon, Université de Lyon, Université Lyon 1, Lyon, France; 3 Facultés de Médecine Est et Lyon Sud, Université de Lyon, Université Lyon 1, Lyon, France; 4 Service d'Anatomie et de Cytologie Pathologiques, Hôpital Lyon-Sud, Hospices Civils de Lyon, Pierre Bénite, France; 5 AltraBio, Lyon, France; 6 Service d'Urologie, Hôpital Lyon-Sud, Hospices Civils de Lyon, Pierre Bénite, France; 7 Unité Médicale d'Oncologie Moléculaire et Transfert, Hôpital Lyon-Sud, Hospices Civils de Lyon, Pierre Bénite, France; II Università di Napoli, Italy

## Abstract

**Background:**

Several data favor androgen receptor implication in prostate cancer initiation through the induction of several gene activation programs. The aim of the study is to identify potential biomarkers for early diagnosis of prostate cancer (PCa) among androgen-regulated genes (ARG) and to evaluate comparative expression of these genes in normal prostate and normal prostate-related androgen-sensitive tissues that do not (or rarely) give rise to cancer.

**Methods:**

ARG were selected in non-neoplastic adult human prostatic epithelial RWPE-1 cells stably expressing an exogenous human androgen receptor, using RNA-microarrays and validation by qRT-PCR. Expression of 48 preselected genes was quantified in tissue samples (seminal vesicles, prostate transitional zones and prostate cancers, benign prostatic hypertrophy obtained from surgical specimens) using TaqMan® low-density arrays. The diagnostic performances of these potential biomarkers were compared to that of genes known to be associated with PCa (i.e. PCA3 and DLX1).

**Results and Discussion:**

By crossing expression studies in 26 matched PCa and normal prostate transitional zone samples, and 35 matched seminal vesicle and PCa samples, 14 genes were identified. Similarly, 9 genes were overexpressed in 15 benign prostatic hypertrophy samples, as compared to PCa samples. Overall, we selected 8 genes of interest to evaluate their diagnostic performances in comparison with that of PCA3 and DLX1. Among them, 3 genes: CRYAB, KCNMA1 and SDPR, were overexpressed in all 3 reference non-cancerous tissues. The areas under ROC curves of these genes reached those of PCA3 (0.91) and DLX1 (0.94).

**Conclusions:**

We identified ARG with reduced expression in PCa and with significant diagnostic values for discriminating between cancerous and non-cancerous prostatic tissues, similar that of PCA3. Given their expression pattern, they could be considered as potentially protective against prostate cancer. Moreover, they could be complementary to known genes overexpressed in PCa and included along with them in multiplex diagnostic tools.

## Background

Prostate cancer (PCa) is in men the most prevalent cancer and the second-leading cause of death [1]. Current diagnosis is based on the histological examination of prostate needle-core biopsies. Increased serum PSA (prostate specific antigen) is widely used by physicians, although not specific, for deciding prostate biopsies and detecting prostate cancer [2]. In fact, benign prostatic hypertrophy (BPH) and other non-cancerous prostate conditions, such as acute or chronic prostatitis, can raise PSA levels. This leads to unnecessary prostate biopsies since more than 60% of biopsies suggested by PSA test ultimately turn up negative. Furthermore, PSA test does not differentiate clinically significant from indolent tumors, resulting in overdiagnosis and sometimes overtreatment. There is consequently a need for novel biomarkers that aid clinical decision making about biopsy and initial treatment.

The usual strategy for cancer biomarker discovery is to compare prostate cancer with benign prostate tissue. Thus was identified the promising biomarker PCA3 (prostate cancer gene 3) by differential display comparing cancer with normal and benign hyperplasia prostate specimens [3]. High-throughput technologies, such as microarray analysis and mass spectrometry, have boosted the field of prostate cancer biomarker discovery. Since the first publications in the end of the 90 s and the beginning of the 2000 s, many biomarkers or “signature” profiles specific to each pathologic state, e.g. normal *versus* cancer, have been proposed for prostate cancer diagnosis (revue in [4], [5]). Whether these potential new biomarkers are all clinically relevant remains nevertheless uncertain since none reach the development phase of PCA3 [6].

Prostate is one of the androgen-sensitive tissues. More specifically, both embryonic development of prostate and prostate maintaining at adulthood are dependent on a normal tissue impregnation by androgens. Androgens act through a specific receptor, AR (androgen receptor), which belongs to the nuclear receptor superfamily. AR is involved in PCa growth [7], [8] but also in its initiation [9], through the induction of several genes [10], [11], [12], [13]. Whether these genes can be considered as potential biomarkers for early diagnosis of prostate cancer deserves to be evaluated. We therefore proposed a two-steps strategy for the purpose of prostate cancer diagnosis biomarker discovery. We first hypothesized that potential biomarkers for early diagnosis of prostate cancer could be identified among androgen-regulated genes (ARGs). We selected ARGs in immortalized RWPE-1 epithelial prostate cells stably expressing AR [14], using RNA microarrays and validation by qRT-PCR. Second, we evaluated comparative expression of these ARGs in normal prostate and normal prostate-related androgen-sensitive tissues that do not (or rarely) give rise to cancer. We used matched samples of seminal vesicles, prostate transitional zones and prostate cancers from patients operated on for radical prostatectomies and validated their diagnostic performances by demonstrating their ability to discriminate between normal prostate, BPH and cancer tissues, and comparing it with that of known biomarkers of prostate cancers (PCA3, DLX1).

## Methods

### Transcriptomic analysis on RWPE-1-AR cells stimulated by R1881

We used the stable cell line RWPE-1-AR that constitutively expresses an exogenous AR as described elsewhere [14]. Cells were maintained in keratinocyte growth medium (Invitrogen 17005-042) supplemented with rEGF (recombinant epithelial growth factor) and BPE (bovine pituitary extract) (Invitrogen 37000015), antibiotics and antimycotics. RWPE-1-AR cells were stimulated with the non-metabolisable androgen, R1881 (10–9 M), in the growth medium deprived of BPE. Three independent cell culture experiments for each treatment condition (vehicle or R1881 for 3 h and 24 h) were performed for microarray analysis. Total RNA was extracted using the RNeasy® mini kit (74104, Qiagen). The RNA concentration was measured by OD reading using a Nanodrop spectrophotometer.

To check the response to R1881 in the stimulated cells, the expression of a panel of known target AR genes was analyzed by quantitative polymerase chain reaction (qPCR) for each condition. The cDNA obtained from 1 μg retrostranscribed RNA (Promega M1701) was amplified using QuantiTect SYBR® Green PCR Kit (Qiagen 204145). Primers provided from Qiagen: Hs_KLK3/PSA (QT00027713), MME (QT00048755), Hs_TMPRSS2 (QT00058156), Hs_MMP2 (QT00088396), Hs_MCM10 (QT00030338), and Hs_TPB (QT00000721) as housekeeping gene.

The quality of extracted RNA was assessed using a Bioanalyzer 2100 (Agilent technologies). RNA integrity numbers of all samples were 10. Reverse transcription, labeling and hybridization on Affymetrix Human 133 plus 2.0 Arrays were performed by ProfileXpert service (Bron, France) according to Affymetrix™ protocols (Expression Analysis Technical Manual, 2008, Affymetrix). One μg of total RNA was used for preparation of biotinylated cRNA and 15 μg of cRNA were hybridized. The Affymetrix Fluidics Station 450 was used for washing and staining. Arrays were scanned using the GeneChip Scanner 3000 (Affymetrix). Affymetrix CEL files were analyzed in R using the Bioconductor suite of packages. Raw probe signals were background corrected, normalized and summarized using the RMA procedure. Linear models were applied using the limma package in order to identify genes with potentially significant change in expression in response to time effect or R1881 treatment at each duration (model formula: ∼ Duration + Duration:R1881). The empirical Bayes method was used to compute moderated p-values that were then corrected for multiple comparisons using the Benjamini and Hochberg's false discovery rate (FDR) controlling procedure. The microarray data have been deposited and described, in accordance with MIAME guidelines, in Gene Expression Omnibus under the accession number GSE29232 (http://www.ncbi.nlm.nih.gov/geo/query/acc.cgi?acc=GSE29232).

To assess that the relative RNA expression levels of 14 regulated-transcripts were consistent with the microarray data, RT-qPCR was performed in triplicates from the same RNA as in microarray experiments using QuantiTect SYBR® Green PCR Kit (Qiagen 204145). Primers provided from Qiagen: Hs_EGFR (QT00085701), SOX2 (QT00237601), Hs_NDRG1 (QT02290232), Hs_MME (QT00048755), Hs_TFPI2 (QT00062804), Hs_CDK5R1 (QT00231644), Hs_SCNN1G (QT00063217), Hs_RHOB (QT00227409), Hs_PRDM1 (QT00060494), Hs_IL7R (QT00053634), Hs_SERPINB2 (QT00077497), Hs_PAX9 (QT00023317), Hs_FST (QT01665853), Hs_ADAMTS1 (QT00098588) and Hs_TPB (QT00000721) as housekeeping gene to normalize raw data. Correlations between microarray and qPCR results were performed using Spearman test and linear regression analysis.

### Identification of candidate biomarker genes

Potential candidate genes were selected with the following criteria: 1) androgen-regulated: considering log2 fold-change cut off value to 1.2 and the FDR (false discovery rate) with *P* <0.05; 2) expressed at significantly higher levels after treatment (3 h and/or 24 h duration); 3) Gene Ontogeny categories and relevant pathways using Ingenuity Pathway Analysis (IPA®) software (Ingenuity Systems). A total of 36 gene targets were chosen for confirmation of (differential) expression in patient samples.

### Tissue specimens

#### Ethics statement

Non-interventional biomedical research protocol for tissue samples conservation after a prostate surgery has been set-up at the Centre Hospitalier Lyon-Sud and the Ethics Committee in Lyon (CPP Sud-Est 2) specifically approved it for this study. Therefore, patients admitted to the urology department in the Centre Hospitalier Lyon-Sud were informed and gave voluntary, signed informed consent prior to any tissue sample conservation and for research use.

#### Tissue samples

Prostate cancer (PCa), prostate transition zone (PTZ) and seminal vesicle (SV) frozen tissues were obtained from radical prostatectomies. The Gleason score (GS) is used for prostate cancer tissue grading. The pTN pathological tumor staging was determined according to UICC TNM classification 2002 (6^th^ edition). Frozen tissues from patients with BPH were obtained from transurethral resection of the prostate. The absence of cancer in BPH specimens was assessed by a pathologist experienced in the field of prostate diseases. The pathological characteristics of the cancer patients included in the study are summarized in Table 1.

**Table 1:** Pathological characteristics of cancer patients included in the study.

Available tissues			pTN	Gleason score (GS)at radical prostatectomy	
PCa	PTZ	SV			
X	X	X	pT3aN1	8	4+4
X	X	X	pT3aN1	8	4+4
X	X	X	pT3aN0	7	3+4
X	X	X	pT2cN0	7	4+3
X	X	X	pT2cN0	7	3+4
X	X	X	pT2cN0	7	3+4
X	X	X	pT3aN0	7	4+3
X	X	X	pT3bN0	7	4+3
X	X	X	pT2cN0	7	3+4
X	X	X	pT2cN0	7	4+3
X	X	X	pT3bN0	9	4+5
X	X	X	pT3aN1	9	5+4
X	X	X	pT2cN0	7	4+3
X	X	X	pT3aNx	7	3+4
X	X		pT3aN0	7	3+4
X	X		pT3aN0	7	4+3
X	X		pT2bN0	7	3+4
X	X		pT2bNx	7	3+4
X	X		pT3aN0	8	4+4
X	X		pT3aN1	9	4+5
X	X		pT3bN1	7	4+3
X	X		pT3bN0	7	4+3
X	X		pT2cN0	7	4+3
X	X		pT2cN0	7	3+4
X	X		pT3bN0	7	4+3
X	X		pT3bN0	8	4+4
X*	X*		pT3aN0	8	4+4
X*	X*		pT3bN1	7	4+3
X		X	pT3aN0	7	3+4
X		X	pT3bN0	7	4+3
X		X	pT3bN1	9	4+5
X		X	pT2cN0	7	4+3
X		X	pT3bN1	7	4+3
X		X	pT3bN0	9	4+5
X		X	pT3bN0	7	4+3
X		X	pT3aN0	7	4+3
X		X	pT3aN0	9	4+5
X		X	pT3bN1	7	4+3
X		X	pT3aN0	7	3+4
X		X	pT3aN0	7	4+3
X		X	pT3bN1	7	4+3
X		X	pT3bN0	7	4+3
X		X	pT3aN0	6	3+3
X		X	pT3bN0	7	4+3
X		X	pT3aN0	7	3+4
X		X	pT3bN1	7	4+3
X		X	pT3aN1	9	5+4
X		X	pT3bN0	7	4+3
X		X	pT3bN0	8	4+4
X*			pT3bN0	7	4+3

PCa = Prostate cancer; SV = Seminal vesicle; PTZ = Prostate Transition Zone. *: tissue samples added for the ROC curves construction. The pTN pathological tumor staging was determined according to UICC TNM classification 2002 (6^th^ edition). For comparative expression studies, tissues samples for PCa, PTZ and SV were obtained from the same surgical specimens.

For comparative expression studies in PCa, SV and PTZ matched tissue samples, that is obtained from the same surgical specimens, the absence of cancer cells in SV and PTZ was checked by rigorous histological examination of the tissues adjacent to those pieces reserved for frozen and RNA extraction. For diagnostic purposes, were used 50 PCa tissue samples: GS 6 (*n = 1*), GS 7(3+4) (*n = 12*), GS 7(4+3) (*n = 24*), GS 8 (*n = 6*), and GS 9 (*n = 7*).

### RNA extraction and reverse transcription

Frozen tissues were stored in liquid nitrogen until RNA extraction Total RNA was extracted from homogenized tissues by Trizol® (Invitrogen) and was assessed for integrity using the 2100 Bioanalyzer® (Agilent). 1 μg of total RNA was converted to cDNA using the First-Strand cDNA Synthesis kit® (Invitrogen). The resulting cDNA was used immediately for real-time qPCR.

### TLDA real-time qPCR

The Applied Biosystems TaqMan technology (TaqMan® low-density arrays: TLDAs) is used for the quantitative analysis of gene expression. The principle of the high-throughput real-time qPCR system is based on the 384-well microfluidic card (8 separate loading ports, each with 48 separate wells) [15]. Each 2-μl well contains specific, user-defined primers and probes, capable of specifically detecting a single gene. Each cDNA sample (100 ng cDNA equivalent) was added to an equal volume of 2× TaqMan Gene. Expression Master Mix® (Applied Biosystems) for a total of 100 μl per port. After gentle mixing and centrifugation, the mixture was transferred to a loading port on a TLDA card. The array was centrifuged twice for 1 min, each at 1200 rpm, to distribute the samples from the loading port to each well. The card was then sealed and PCR amplification performed using a 7900HT Fast Real-time PCR System® (Applied Biosystems) following the protocol described by the manufacturer.

In this study, the mRNA levels of 48 genes were measured. Each plate had 18S-specific primer/probe as an endogenous control. In addition, HMBS, RPL13A, ACTB, TBP, and UBB were measured as potential housekeeping genes. Two technical duplicates were performed for each different sample.

### Statistical analysis

#### Real-time data

The threshold cycle (Ct) was automatically given by the SDS2.2® software package (Applied Biosystems). Relative quantities (RQ) were determined using the equation: RQ = 2-ΔΔCt. All data were generated in duplicate for each gene expression per sample. The TaqMan Array Cards data were simultaneously analyzed for differential expression using the Integromics RealTime StatMiner 4.0® package which integrates BioConductor R software. The different steps of the workflow process of PCR data analysis: quality control, selecting the most stable endogenous control, data normalization and relative quantification, were achieved with this bioinformatic tool. As controls for the presence of prostate cells in the prostatic samples, two prostate-specific markers, members of the kallikrein family, i.e. KLK2 and KLK3/PSA, were used for normalization. Fold changes in gene expression were presented by Log10RQ (Log10RQ = 0 if no expression change; Log10RQ = 1 if the test sample is expressed 10 times greater than in the calibrator sample; Log10RG = −1 if the test sample is expressed 10 times less than in the calibrator sample). The Student's t paired test was used for comparisons between matched samples. The nonparametric Wilcoxon test was used for comparisons between non-matched samples. The Benjamini and Hochberg False Discovery Rate was taken as the level of significance. Significance was considered as *P*<0.01.

#### Evaluation of diagnostic performances

Receiver-operating characteristic (ROC) curves were calculated in order to assess the diagnostic power of each separate variable univariately by the area under the curve (AUC) of the ROC curve. Values for the ROC curves were -ΔCt normalized using the geometric mean of TBP, KLK2 and KLK3. The 95% confidence interval (95% IC) of the AUC values were calculated as described (13). For this expression study, potential biomarkers were considered valuable if AUCs ≥0.9 (13). All these statistical calculations were performed using STATA®11.0 software (College Station, Texas).

## Results and Discussion

### Androgen-regulated genes expression profiles and identification of potential mRNA biomarkers

We aimed to identify androgen-dependent gene activation programs potentially involved in the early stages of prostate carcinogenesis. We therefore decided to use a cell model as close as possible to normal prostate cells rather than prostate cancer cell lines, in which molecular events are likely to represent late stages of prostate cancer development. We used non-cancerous RWPE-1 cells previously obtained by immortalization of non-neoplastic adult human prostatic epithelial cells with human papillomavirus 18 [16]. Despite immortalization, these cells proved to behave like nearly normal prostate cells: conservation of Y chromosome, normal expression of cytokeratins and E-cadherin, growth stimulation as well as PSA and AR expression in response to androgens [17], [18]. They also retained the ability to develop, particularly in Matrigel 3D cultures, well-polarized hollow spheroids undergo and eventually acinar differentiation as a response to growth factors and as a result of interactions with extra-cellular matrix [17], [18], [19].

To reinforce androgen-dependent gene activation programs, we used stably transfected RWPE-1 cells with the wild-type AR gene [14]. The resulted RWPE-I-AR cells were strongly androgen-sensitive as checked by proliferation assays. These cells were treated by the non metabolisable synthetic androgen R1881 for 3 h and 24 h. Androgen- induced stimulation was first checked by quantitative RT-PCR of a panel of known target AR genes including KLK3/PSA, MME (macrophage metalloelastase), and TMPRSS2 (data not shown). Extracted cDNAs from cells treated or not with R1881 were then hybridized on Affymetrix Human 133 plus 2.0 arrays.

Transcriptomic profiling identified, using a log2 fold-change cut off value of 1.2, 75 genes exhibiting up-regulation and 33 genes exhibiting down-regulation in R1881-treated RWPE-1-AR cells during 3 h relative to controls (vehicle treated), considering the FDR (false discovery rate) with *P*<0.05. Transcriptomic profiling identified 208 genes exhibiting up-regulation and 116 genes exhibiting down-regulation in R1881-treated RWPE-1-AR cells during 24 h relative to controls, considering the FDR with *P*<0.05.

Validation experiments were performed to confirm the accuracy of array gene expression measurements on selected transcripts differentially expressed at 3 h and/or 24 h duration R1881 treatment (7 up-regulated and 7 down-regulated) using LightCycler real-time qPCR: EGFR, SOX2, NDRG1, MME, TFPI2, CDK5R1, SCNN1G, RHOB, PRDM1, IL7R, SERPINB2, PAX9, FST, and ADAMTS1. The results confirmed that the relative RNA expression levels were consistent with the microarray data (Table S1).

To identify and prioritize biomarker candidates among genes found to be differentially expressed after 3 h and/or 24 h R1881 treatment, we based *in silico* analyses on biological characteristics considered to be the most relevant in both literature survey and Ingenuity Pathway Analysis (IPA®). Analyses with this software returned the following information: 1/ the relevant functions associated with the dataset and 2/ affected signaling and metabolic pathways associated with the dataset. In addition to high fold-change in microarray dataset at 3 h and/or 24 h of R1881 exposition, genes were designed as of interest if they have at least one these functional annotations or disease associations in the Ingenuity Knowledge database: possible detection in urine, well-known tissue expression and, in particular, differential expression in prostate cancer cell lines, in prostate benign tissues and/or in prostate cancer, and/or strong association with carcinogenetic processes. We also favored up-regulated genes to down-regulated ones in an attempt to facilitate potential future use in clinical practice. Thirty six genes matched these criteria (Table 2) and were used for further study.

**Table 2:** Genes represented on Taqman array, including official gene symbols and Applied gene expression assay ID.

Gene name	Symbol	Assay ID
***Housekeeping genes***		
18S rRNA	18S	Hs99999901 s1
Actin beta	ACTB	Hs99999903 m1
Hydroxymethylbilane synthase	HMBS	Hs00609297 m1
Ribosomal protein l13a	RPL13A	Hs01926559 g1
TATA box binding protein	TBP	Hs00920497 m1
Ubiquitin B	UBB	Hs00430290 m1
***Microarray-based selected genes***		
Aldo-keto reductase family 1, member C1/2	AKR1C1/AKR1C2	Hs00413886 m1
Aldo-keto reductase family 1, member C3	AKR1C3	Hs00366267 m1
Activated leukocyte cell adhesion molecule	ALCAM	Hs00233455 m1
Aquaporin 3 (Gill blood group)	AQP3	Hs00185020 m1
Autism susceptibility candidate 2	AUTS2	Hs00322711 m1
BCL2/adenovirus E1B 19kda interacting protein 3	BNIP3	Hs00969293 mH
Chromosome 13 open reading frame 15	C13orf15	Hs00204129 m1
CD24 molecule / CD24 molecule-like 4 pseudogene	CD24L4 CD24	Hs00273561 s1
Cyclin-dependent kinase 5, regulatory subunit 1 (p35)	CDK5R1	Hs00243655 s1
Claudin 7	CLDN7	Hs00600772 m1
Claudin 8	CLDN8	Hs00273282 s1
CNKSR family member 3	CNKSR3	Hs00295109 m1
Crystallin, alpha B	CRYAB	Hs00157107 m1
Dapper, antagonist of beta-catenin, homolog 1 (Xenopus laevis)	DACT1	Hs00420410 m1
Delta-like 1 (Drosophila)	DLL1	Hs00194509 m1
Fibronectin leucine rich transmembrane protein 3	FLRT3	Hs00183798 m1
Forkhead box A2	FOXA2	Hs00232764 m1
Follistatin	FST	Hs00246260 m1
Hydroxyprostaglandin dehydrogenase 15-(NAD)	HPGD	Hs00168359 m1
Potassium large conductance calcium-activated channel, subfamily M, alpha member 1	KCNMA1	Hs01119498 m1
Laminin, alpha 3	LAMA3	Hs00165042 m1
Lysyl oxidase	LOX	Hs00942480 m1
Membrane metallo-endopeptidase	MME	Hs00153519 m1
N-myc downstream regulated 1	NDRG1	Hs00608389 m1
PR domain containing 1, with ZNF domain	PRDM1	Hs00153357 m1
RAS, dexamethasone-induced 1	RASD1	Hs00607394 g1
Regulator of G-protein signaling 2, 24kda	RGS2	Hs00180054 m1
Ras homolog gene family, member B	RHOB	Hs00269660 s1
Sciellin	SCEL	Hs01557103 m1
Sodium channel, nonvoltage-gated 1, gamma	SCNN1G	Hs00168918 m1
Serum deprivation response	SDPR	Hs00190538 m1
Selenoprotein P, plasma, 1	SEPP1	Hs00193657 m1
Serpin peptidase inhibitor, clade B (ovalbumin), member 1	SERPINB1	Hs00229084 m1
Serum/glucocorticoid regulated kinase 1	SGK1	Hs00985033 g1
Serglycin	SRGN	Hs01004159 m1
Tissue factor pathway inhibitor 2	TFPI2	Hs00197918 m1
***Prostate cancer biomarkers***		
Prostate cancer antigen 3 (non-protein coding)	PCA3	Hs01371938 m1
Distal-less homeobox 1	DLX1	Hs00698288 m1
Transmembrane protease, serine 2	TMPRSS2	Hs00237175 m1
***Prostate-specific genes***		
Kallikrein-related peptidase 2	KLK2	Hs00428383 m1
Kallikrein-related peptidase 3	KLK3	Hs03063374 m1
***Marker of androgen sensitivity***		
Androgen receptor	AR	Hs00907244 m1

### Quantitative RT-PCR analysis of human cancerous and non-cancerous prostate matched tissues

To further investigate the expression levels of androgen regulated genes identified by microarray analysis, quantitative RT-PCR analysis was performed on the selected targets using human PCa and prostate transition zone (PTZ) matched tissues. PTZ was chosen because it is known to rarely give rise to prostate cancer [20]. Our goal was to identify biomarkers that can distinguish between cancerous and non-cancerous prostatic tissues. We chose large-scale RT-PCR RNA quantification on TaqMan® low-density arrays (TLDAs; Applied Biosystems) that allow, per sample, simultaneous analyses of up to 48 targets on customized cards. Quantitative RT-PCR was chosen as a base for comparison because it allows mRNA quantification, a process previously used to assess tissue expression [21], [22], [23], [24], [25], [26] and urinary amounts [22] of PCA3 (prostate cancer gene 3), a gene we wished to use as a control positive gene. PCA3 is indeed now recognized as a valuable biomarker of prostate cancer (revue in [27], [28]) and has been shown to be specifically expressed in up to 95% of PCa [3], [29]. PCA3 was therefore used as a positive control, reflecting PCa- specific expression panel. TMPRSS2 [30] and DLX1 [31], [32] are also potential PCa biomarkers, as disclosed by examining microarray data using the Oncomine database, and their expression was similarly evaluated as positive controls. In addition, we evaluated expression of 6 potential housekeeping genes: 18S, ACTB, HMBS, RPL13A, TBP, and UBB, as well as 2 genes considered as prostate-specific genes: KLK2 and KLK3/PSA. As a global marker of androgen-sensitive tissues (prostate and seminal vesicles), we also selected AR gene for analysis (Table 2).

Whether PCa and PTZ tissues express similar RNA amounts is unknown. Normalization by a housekeeping gene was therefore warranted. Recent reports showed that housekeeping genes can in fact present serious differences between tissues, cell lines or clinical samples [33]. We therefore first sought to determine which potential housekeeping genes were stably expressed under the different conditions from among the 6 we chose. TBP was found as the most stable endogenous control using the NormFinder algorithm of the StatMiner® package and was used as the normalization gene in the following parts of the study. The prostate-specific genes KLK2 and KLK3/PSA also disclosed clear expression stability and were used together with TBP as the endogenous control (geometric mean).

**Figure 1:**
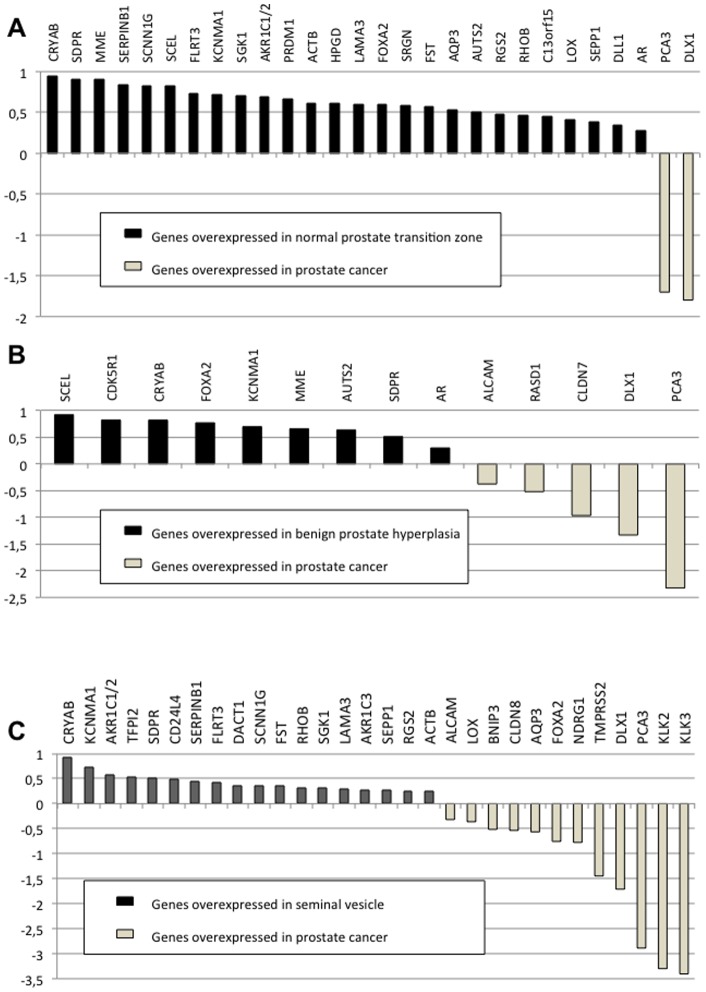
Log10RQ bar chart of significant expression changes. Y-axis: log10 of RQ (relative quantities); X-axis: genes whose expression was statistically significantly different between: A) 26 matched normal prostate transition zone and prostate cancer samples B) 27 prostate cancer samples and 15 samples of benign prostatic hyperplasia C) 35 matched seminal vesicle and prostate cancer samples.

We compared expression of all the selected genes in **26 matched normal prostate PTZ and PCa tissues**, obtained from radical prostatectomy specimens. Accordingly, 26 genes were found to be significantly (*P*<0.01) overexpressed in the normal prostate transition zone as compared to PCa (Figure 1A and Figure S1). Ingenuity Pathway Analysis showed that 17 and 21 of these 26 genes belong to 2 significantly represented biological functions: cellular movement (migration of cells in particular) and cancer (epithelial carcinoma in particular), respectively. Conversely, only PCA3 and DLX1 genes were determined to have higher level (more than 50- and 60-fold, respectively) in PCa tissues as compared to the matched normal PTZ tissues (*P*<0.01) (Figure 1A). It is worth noting that ACTB (encoding beta-actin), usually considered as a potential housekeeping gene, was also found to be overexpressed in PTZ. In fact, ACTB is androgen-regulated [34], [35] and has previously reported to be differentially expressed between cancerous and non-cancerous prostate tissues [36].

Benign prostate hypertrophy (BPH) is an interesting model to which comparison with PCa is pertinent. These two pathological conditions indeed share several points including specific local environment (same blood supply and exposition to mitogens), androgen-sensitivity and uncontrolled cell proliferation. By contrast to PCa, BPH originates from PTZ and is thought to never be the source of malignant transformation. Expression of the selected genes was therefore compared **in the 26 previously tested PCa specimens and in 15 unrelated BPH samples**, in which the absence of cancerous cells was ascertained by a pathologist expert in the prostatic pathology field. We used the same 3 normalization genes (TBP, KLK2 and KLK3/PSA), which proved to be stable according to NormFinder algorithm. In this way, we identified 9 genes significantly (*P*<0.01) overexpressed in BPH as compared to PCa (Figure 1B and Figure S2): AR, AUTS2, CDK5R1, CRYAB, FOXA2, KCNMA1, MME, SCEL, and SDPR. CDK5R1 (and only this gene) was also found to be overexpressed in BPH tissues when comparing to its expression in normal prostate PTZ. It encodes the so-called p35 protein, which acts as an essential activator of CDK5, a strong regulator of neuronal migration. Protein p35 is weakly expressed in PCa tissue samples and proved to be involved in prostate cell apoptosis [37]. To the best of our knowledge, specific expression in BPH has never been reported. Five genes were identified as overexpressed in PCa as compared to BPH: ALCAM, CLDN7, DLX1, PCA3 and RASD1 (Figure 1B). The PCA3 gene, highly specific to prostate cancer cells, has been reported to be overexpressed 66 to 100-fold in prostate cancer *versus* normal prostate and 140-fold in prostate cancer *versus* BPH [3], [21]. In our study, we observed a strong overexpression in PCa from 50 to 210 fold, as compared to normal prostate and BPH, respectively. DLX1 showed the same expression pattern, as previously observed [31]. ALCAM and CLDN7 both encode proteins localized to the inter-cellular junctions of diverse epithelia and have previously been suggested as androgen-regulated and overexpressed in PCa [38], [39]. RasD1 was initially described as a dexamethasone-inducible ras-related protein and potential actor in preventing aberrant cell growth in several cell lines [40]. According to Ingenuity Pathway Analysis®, among the 14 genes thus found to be differentially expressed in BPH and prostate cancer, all were involved in a significantly represented functional network – cell growth and proliferation – except PCA3 (little is known about its function, but PCA3 in the control of PCa cell survival, in part through modulating AR signaling [41]) and CDK5R1 (rather involved, along with 8 other genes, in a significantly represented biological function: cell death and survival).

### Quantitative RT-PCR analysis of human cancerous prostate and seminal vesicle matched tissues

The above comparative expression studies allowed us to identify genes overexpressed in non cancerous prostate tissues as compared to prostate cancer. Despite their potential diagnostic value, these genes may also be of importance as putative markers of processes protective against cancer transformation. Their high expression in BPH and normal PTZ could be related to the poor or null incidence of cancer in these tissues, while their weak expression in the prostate could allow cancer initiation with great frequency. Such a mechanism has been recently suggested in another prostate-related tissue: seminal vesicle [42]. The authors used a 2-step strategy by first selecting genes with significantly higher expression levels in seminal vesicles rather than in normal prostate and secondly by crossing this list with genes identified elsewhere because their expression was silenced in PCa by promoter DNA methylation. Eight genes of interest were therefore identified [42]. The present study is consistent with this published one in that we also used a 2-step strategy that allowed us to successfully select candidate androgen-regulated genes whose expression was measured in PCa samples and compared to reference prostate tissues known to infrequently or never give rise to cancer despite their common embryologic origin and carcinogenic exposure. Likewise, it is known that, despite features shared in common by the prostate and the seminal vesicles, including especially androgen-dependant growth and function, the incidence of cancer of the seminal vesicles and prostate gland is strikingly different [42]. Importantly, the basis of this disparity could be correlated with mechanisms underlying prostate carcinogenesis [42]. Adding tissues from normal seminal vesicles was therefore considered in the present study, to reinforce expression comparison between prostate cancer and a reference benign tissue, highly protected against cancer transformation.

We therefore evaluated gene expression in **35 matched seminal vesicle and PCa tissues** using the same selected genes (Table 2). The lack of seminal vesicle involvement by prostate cancer was carefully pathologically ascertained before use in the study. TBP was found as the most stable endogenous control using the NormFinder algorithm of the StatMiner® package and was used as the normalization gene. As expected, the prostate-specific KLK2 and KLK3 genes were underexpressed in seminal vesicle as compared to prostate tissue (*P*<0.01). Ten other genes were also significantly overexpressed in PCa as compared to seminal vesicle: ALCAM, LOX, BNIP3, CLDN8, AQP3, FOXA2, and NDRG1, as well as the expected TMPRSS2, DLX1 and PCA3 (Figure 1C and Figure S3). By contrast, 18 genes were found to be significantly overexpressed in seminal vesicle as compared to PCa: CRYAB, KCNMA1, AKR1C1/2, TFP12, SDPR, CD24L4, SERPINB1, FLRT3, DACT1, SCNN1G, FST, RHOB, SGK1, LAMA3, AKR1C3, SEPP1, RGS2 and ACTB (*P*<0.01). Similarly to PTZ *versus* prostate cancer comparison, Ingenuity Pathway Analysis showed that cellular movement and cancer were the 2 significantly represented biological functions.

Overexpressed in seminal vesicle, CRYAB, KCNMA1 and SDPR were also overexpressed in both normal PTZ and BPH. All 3 have previously been found weakly expressed in PCa but none of them has been shown to be androgen-regulated or involved in prostate carcinogenesis [43], [44], [45].

### Comparison of diagnostic performances of candidate PCa biomarker genes with PCA3 biomarker

We next attempted to compare the diagnostic potential of the genes previously found as differentially expressed in discriminating cancerous and non-cancerous tissues, with that of the well-known PCA3 biomarker. PCA3 is a long non-coding RNA highly expressed in PCa cells. RNA quantification of PCA3 from prostate cells is indeed routinely performed in urine samples to help clinicians in guiding biopsy decision [27], [28]. We used the PCa tissue cohort (n = 50) and compared it to the cohort of non cancerous prostate tissues (n = 44, i.e. 28 PTZ and 16 BPH). We first chose for evaluation the 3 genes found to be overexpressed in all 3 benign tissues (seminal vesicle, normal PTZ and BPH) as compared to PCa: CRYAB, KCNMA1 and SDPR. We also favored the DLX1 gene, since we found it to be always overexpressed in prostate cancer tissues. Other genes also significantly overexpressed in PCa as compared to other tissues were also evaluated: ALCAM, RASD1 and CLDN7. The AKR1C1/2 gene was also considered because of its functional role in androgen bioavailability (20). At last, CDK5R1 was overexpressed in BPH as compared to PCa, but also when comparing BPH to seminal vesicle and normal PTZ (data not shown). As a potential specific marker of BPH, it was therefore also included in the evaluation of diagnostic potential since BPH constitutes the most frequent diagnostic challenger to prostate cancer in the context of elevated serum PSA.

**Figure 2:**
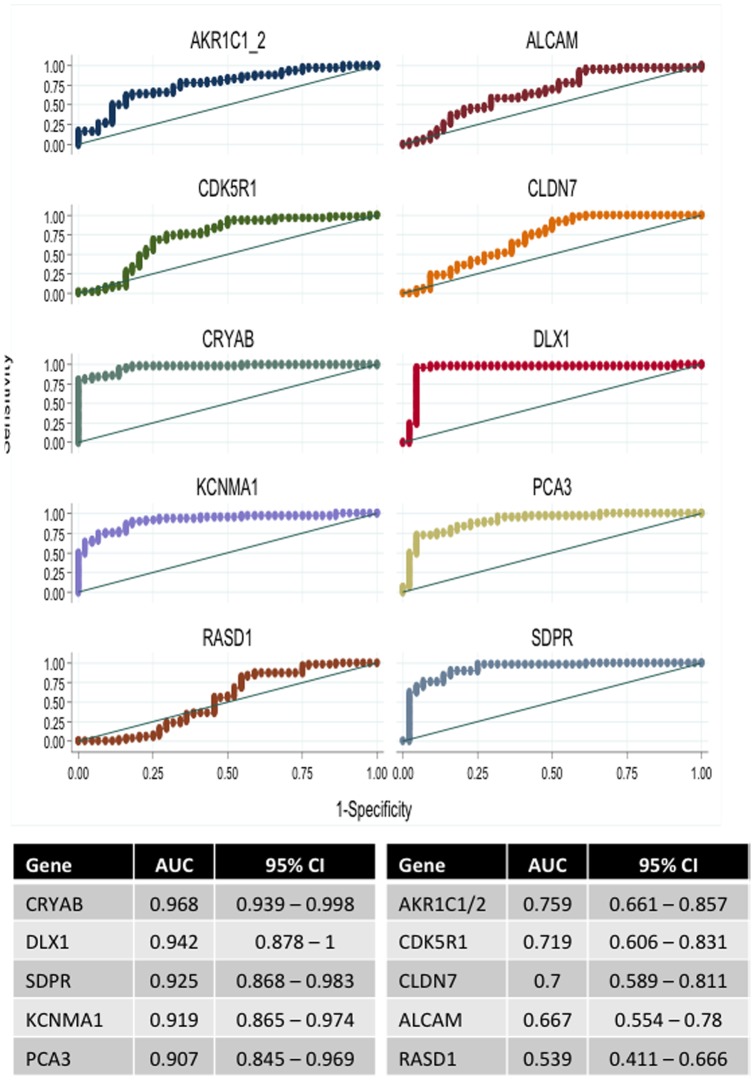
ROC curves of genes differentially expressed between cancerous and non cancerous prostate conditions. Receiver Operator Characteristic (ROC) curves evaluated discriminatory properties of genes differentially expressed between 50 prostate cancer and 44 non cancerous prostate specimens. Areas under the curves (AUCs) were calculated as a measure of predictive reliability.

Receiver Operating Characteristic (ROC) curves were constructed using normalized Ct values and the areas under curve (AUCs) were calculated (Figure 2). These AUCs represent an appropriate means to evaluate global discriminative properties, an AUC greater than 0.90 being considered as excellent [46]. As expected, PCA3 was found to be highly discriminating, with an AUC of 0.91. This was highly reminiscent of the results obtained in the expression study that accompanied the pioneer report of urine PCA3 test: prostate tumor and normal prostate tissue specimens were analyzed for PCA3 expression and the resulting AUC-ROC value was 0.98 [22]. DLX1 gave similar results (AUC: 0.96), whereas none of the 3 potential prostate cancer-specific genes, ALCAM, RASD1 and CLDN7, gave AUC values higher than 0.9. While AKR1C1/2 and CDK5R1did not, CRYAB, KCNMA1 and SDPR gave significantly high AUCs. Access to Oncomine database (using cancer *vs* normal analysis with PCa as the cancer type) confirmed that all 3 genes have already been found in several expression data sets as significantly underexpressed in prostate cancer when compared to non-cancerous tissues. More specifically, down-regulation of KCNMA1 gene has been related with CpG hypermethylation and suggested to be correlated with prediction of prostate cancer recurrence [45]. Underexpression of SDPR in PCa was also found in another study [44]. Interpretation of a test based on a down-regulated marker can be more difficult than that of a test based on an up-regulated marker. However, association of two markers with different kinetics can also be of interest when one is preferentially used for its good positive predictive value (high cancer risk is the up-regulated marker is positive) while the other is used for its good negative predictive value (low cancer risk if the down-regulated marker is positive). We therefore attempted to evaluate whether association of the genes that provided an AUC >0.9 could be of interest. A regression model comprising the 2 up-regulated genes PCA3 and DLX1 and the 3 down-regulated genes CRYAB, KCNMA1 and SDPR indeed provided an AUC at 0.998 (95%CI: 0.995–1.000). Multivariate logistic regression nevertheless disclosed that none of the 5 markers were independent from each other. When only associating PCA3 (the up-regulated gene currently validated in clinical practice) and CRYAB (the down-regulated gene that provided the higher AUC in our study), we obtained similar AUC (0.991; 95%CI: 0.978–1.000) as the comprehensive model's one while both markers proved to be significantly independent predictors (*P*≤0.002).

It is here worthy to recall that the down-regulated genes were selected by a 2-step strategy, the first step being the identification of androgen-responsive genes in a cell model (immortalized prostate cells stably overexpressing AR) as close as possible to normal cells, and the second favoring those genes that are overexpressed in androgen-sensitive tissues that do not or not frequently give rise to prostate cancer. Recently, Thompson et al. indeed recalled that, despite the commune features sharing by the prostate and the seminal vesicles including especially the androgen dependent growth and function, the incidence of cancer of seminal vesicles and prostate gland is strikingly different [42]. They proposed that the bases of this disparity are correlated to mechanisms underlying prostate carcinogenesis [42]. They hypothesized that certain genes highly expressed in seminal vesicles and weakly expressed in the prostate are protective against carcinogenesis. High expression of these genes in seminal vesicles explains the poor incidence of cancer while their weak expression in the prostate allows cancer initiation with great frequency. The same reasoning can be extended to normal transitional zone of the prostate (PTZ), since only 10–20% of PCas arise from transitional zone as compared to the 70–80% that arise from peripheral zone [20]. Another reference tissue is benign prostatic hyperplasia (BPH), which differs from PCa in localization (transitional zone vs peripheral zone) and etiopathogenesis. Both are associated with aging and androgens and show increased number of glandular elements, but BPH cells do not progress to PCa. Elevated expression of tumor-suppressive androgen-responsive genes may be in part responsible for keeping BPH from becoming malignant [47]. Altogether, it can therefore be hypothesized that certain genes overexpressed in seminal vesicle, PTZ and/or BPH as compared to PCa are protective against PCa initiation. CRYAB, KCNMA1 and SDPR have been found that fulfilled these criteria. Because of our two-step strategy, they could represent index of androgen response but they could also be considered as protective against PCa and their decrease can eventually be an effective indicator of early PCa when testing cancerous and non-cancerous prostate tissues.

## Conclusion

We have developed a 2-step strategy that included: 1/ preselection, by microarrays, of androgen-regulated genes in an immortalized prostate cell line (RWPE-1) stably overexpressing AR, and 2/ distinction between those differentially expressed in PCa and androgen-regulated reference tissues infrequently prone to give rise to PCa (transition zone, BPH and seminal vesicle). Among them, CRYAB, KCNMA1 and SDPR were overexpressed in all 3 reference tissues and could be considered as genes protective against PCa and therefore involved in the early stages of prostate carcinogenesis. When compared to a known biomarker of PCa (PCA3), these genes have similar significant diagnostic values for discriminating between cancerous and non cancerous prostatic tissues. They could be complementary to known genes overexpressed in PCa and included along with them in multiplex diagnostic tools.

## Supporting Information

Figure S1 **DCt bar plot: Expression of each tested gene in 26 matched normal prostate transition zone (TZ) and prostate cancer (PCa) samples.** Gene expression is visualized as histograms the height of which represents the mean value of DCt. Error bars represent the standard deviation. All tested genes are represented whether the expression is significantly different in the two conditions or not.
(TIFF)

Figure S2 **DCt bar plot: Expression of each tested gene in 27 prostate cancer (PCa) samples and 15 samples of benign prostatic hyperplasia (BPH).** Gene expression is visualized as histograms the height of which represents the mean value of DCt. Error bars represent the standard deviation. All tested genes are represented whether the expression is significantly different in the two conditions or not.
(TIFF)

Figure S3 **DCt bar plot: Expression of each tested gene in 35 matched seminal vesicle (SV) tissues and prostate cancer (PCa) samples.** Gene expression is visualized as histograms the height of which represents the mean value of DCt. Error bars represent the standard deviation. All tested genes are represented whether the expression is significantly different in the two conditions or not.
(TIFF)

Table S1 **Validation of selected androgen-regulated genes by quantitative PCR.** Results strongly correlated at both treatment by R1881 for 3 h (r coefficient  = 0.977; *p* = 0.0001) and for 24 h (r coefficient  = 0.958; *p* = 0.0001). Relative expression was expressed as fold over the reference group (absence of R1881 treatment). Fc: fold change; SD: standard deviation.
(DOCX)
