# Supplementary figures and images for: Differentially Expressed Androgen-Regulated Genes in Androgen-Sensitive Tissues Reveal Potential Biomarkers of Early Prostate Cancer
Source: PLoS One. 2013 Jun 28;8(6):e66278. doi: 10.1371/journal.pone.0066278 (PMC3696068; doi:10.1371/journal.pone.0066278)

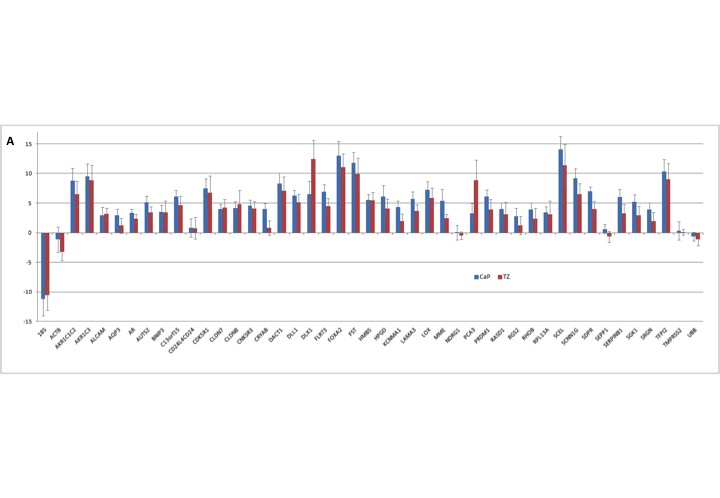

Supplement: Figure S1 — DCt bar plot: Expression of each tested gene in 26 matched normal prostate transition zone (TZ) and prostate cancer (PCa) samples. Gene expression is visualized as histograms the height of which represents the mean value of DCt. Error bars represent the standard deviation. All tested genes are represented whether the expression is significantly different in the two conditions or not. (TIFF) [file pone.0066278.s001.tiff]

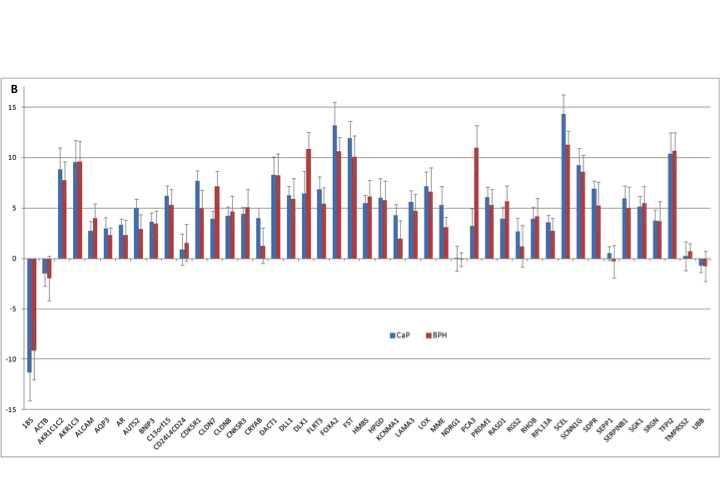

Supplement: Figure S2 — DCt bar plot: Expression of each tested gene in 27 prostate cancer (PCa) samples and 15 samples of benign prostatic hyperplasia (BPH). Gene expression is visualized as histograms the height of which represents the mean value of DCt. Error bars represent the standard deviation. All tested genes are represented whether the expression is significantly different in the two conditions or not. (TIFF) [file pone.0066278.s002.tiff]

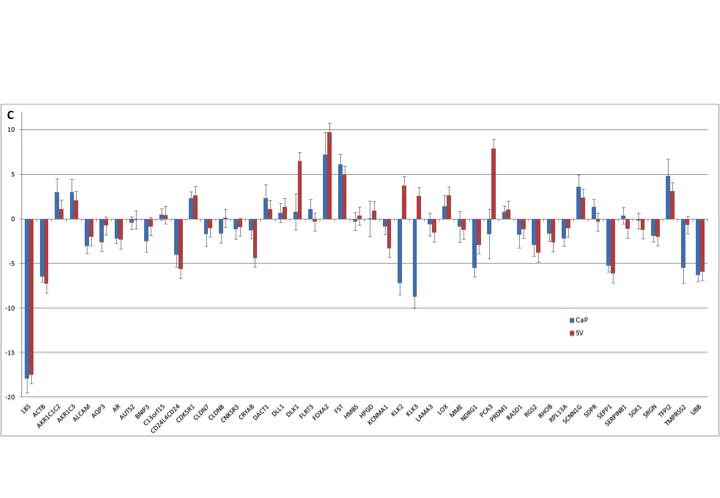

Supplement: Figure S3 — DCt bar plot: Expression of each tested gene in 35 matched seminal vesicle (SV) tissues and prostate cancer (PCa) samples. Gene expression is visualized as histograms the height of which represents the mean value of DCt. Error bars represent the standard deviation. All tested genes are represented whether the expression is significantly different in the two conditions or not. (TIFF) [file pone.0066278.s003.tiff]
