# Supplementary material for: Differentially Expressed Androgen-Regulated Genes in Androgen-Sensitive Tissues Reveal Potential Biomarkers of Early Prostate Cancer
Source: PLoS One. 2013 Jun 28;8(6):e66278. doi: 10.1371/journal.pone.0066278 (PMC3696068; doi:10.1371/journal.pone.0066278)
Supplement: Table S1 — Validation of selected androgen-regulated genes by quantitative PCR. Results strongly correlated at both treatment by R1881 for 3 h (r coefficient = 0.977; p = 0.0001) and for 24 h (r coefficient = 0.958; p = 0.0001). Relative expression was expressed as fold over the reference group (absence of R1881 treatment). Fc: fold change; SD: standard deviation. (DOCX) [file pone.0066278.s004.docx]

| **Gene** | **Fold change observed after 3h treatment by R1881** | | **Fold change observed after 24h treatment by R1881** | |
| --- | --- | --- | --- | --- |
|  | **Microarrays** Fc (*p*-value) | **qRT-PCR** Fc ± SD | **Microarrays** Fc (*p*-value) | **qRT-PCR** Fc ± SD |
| EGFr | -1.78 (NS) | -0.98 ± 0.2 | -2.46 (*p* = 0.019) | -1.71 ± 0.4 |
| Sox2 | -1.78 (*p* = 0.007)) | -1.82 ± 0.8 | -3.48 (*p* < 0.0001) | -6.18 ± 0.8 |
| NDRG1 | 3.03 (*p* < 0.0001) | 1.23 ± 0.59 | 8 (*p* < 0.0001) | 11.34 ± 2.23 |
| MME | 1 (NS) | 1.12 ± 0.15 | 12.12 (*p* < 0.0001) | 15.21 ± 0.62 |
| TFPI2 | 4.29 (*p* < 0.0001) | 4.18 ± 0.63 | 19.7 (*p* < 0.0001) | 35.47 ± 0.83 |
| CDK5R1 | 11.3 (*p* < 0.0001) | 11.42 ± 0.36 | 6.5 (*p* < 0.0001) | 7.46 ± 0.44 |
| SCNN1G | 8 (*p* < 0.0001) | 27.67 ± 4.85 | 12.13 (*p* < 0.0001) | 49.36 ± 13.11 |
| RHOB | 4.6 (*p* < 0.0001) | 4.41 ± 0.87 | 7.46 (*p* < 0.0001) | 7.39 ± 0.95 |
| PRDM1 | 4 (*p* < 0.0001) | 5.96 ± 0.8 | 2.83 (*p* < 0.0001) | 5.06 ± 0.86 |
| IL7R | -2.3 (*p* < 0.0001) | -3.27 ± 0.3 | -3.73 (*p* < 0.0001) | -21.63 ± 0.71 |
| SERPINB2 | -3.25 (*p* < 0.0001) | -4.13 ± 0.41 | -3.48 (*p* < 0.0001) | -3.35 ± 0.1 |
| PAX9 | -4.20 (*p* < 0.0001) | -3.66 ± 1.23 | -2.64 (*p* < 0.0001) | -3.56 ± 0.52 |
| FST | -10.56 (*p* < 0.0001) | -10.8 ± 1.05 | -19.7 (*p* < 0.0001) | -18.53 ± 1.82 |
| ADAMTS1 | -4.92 (*p* < 0.0001) | -6.87 ± 1.88 | -2.46 (*p* < 0.0001) | -3.71 ± 1.33 |

**Table S1: Validation of selected androgen-regulated genes by quantitative PCR**

Results strongly correlated at both treatment by R1881 for 3h (r coefficient = 0.977; *p*= 0.0001) and for 24h (r coefficient = 0.958; *p*= 0.0001). Relative expression was expressed as fold over the reference group (absence of R1881 treatment). Fc: fold change; SD: standard deviation.
